# Estimating Local Structural Equation Models

**DOI:** 10.3390/jintelligence11090175

**Published:** 2023-09-01

**Authors:** Alexander Robitzsch

**Affiliations:** 1 IPN–Leibniz Institute for Science and Mathematics Education, Olshausenstraße 62, 24118 Kiel, Germany; robitzsch@ipn.uni-kiel.de; 2 Centre for International Student Assessment (ZIB), Olshausenstraße 62, 24118 Kiel, Germany

**Keywords:** local structural equation modeling, confirmatory factor analysis, differentiation, dedifferentiation, invariance

## Abstract

Local structural equation models (LSEM) are structural equation models that study model parameters as a function of a moderator. This article reviews and extends LSEM estimation methods and discusses the implementation in the R package sirt. In previous studies, LSEM was fitted as a sequence of models separately evaluated as each value of the moderator variables. In this article, a joint estimation approach is proposed that is a simultaneous estimation method across all moderator values and also allows some model parameters to be invariant with respect to the moderator. Moreover, sufficient details on the main estimation functions in the R package sirt are provided. The practical implementation of LSEM is demonstrated using illustrative datasets and an empirical example. Moreover, two simulation studies investigate the statistical properties of parameter estimation and significance testing in LSEM.

## 1. Introduction

A structural equation model (SEM) is a statistical approach for analyzing multivariate data (Bartholomew et al. 2011; Bollen 1989; Browne and Arminger 1995; Jöreskog et al. 2016; Shapiro 2012; Yuan and Bentler 2007). These models relate a multivariate vector $X=(X_{1},\ldots ,X_{I})$ of observed *I* variables (also referred to as items or indicators) to a vector of latent variables (i.e., factors) η of a dimension smaller than *I*. SEMs constrain the mean vector μ and the covariance matrix Σ of the random variable X as a function of an unknown parameter vector θ. By doing so, the mean vector is constrained as μ(θ), and the covariance matrix is constrained as Σ(θ).

Local structural equation models (LSEM) study SEMs as a function of a univariate moderator variable Hildebrandt et al. (2009, 2016). The moderator variable is the age or time variable in most applications. LSEM has been mentioned as a general tool for assessing measurement invariance across age or other continuous indicators in social sciences (Dong and Dumas 2020; Han et al. 2019; Leitgöb et al. 2023). Note that LSEM has also been abbreviated as LOSEM Briley et al. (2015a, 2015b).

The LSEM method is particularly suited for studying differentiation or dedifferentiation hypotheses (see Hildebrandt et al. 2009 or Molenaar et al. 2010b). Differentiation hypotheses of intelligence and general scholastic abilities describe changes in the relationship between different cognitive abilities (i.e., their structural organization) depending on the level of general ability (ability differentiation), age (differentiation in children and adolescents; dedifferentiation in older adults), and their interaction. Breit et al. (2022) presented a systematic review of 33 reports with data from 51 studies with over 260,000 participants that examined differentiation effects. The findings indicated practically significant ability differentiation in children and adults, and significant age dedifferentiation in older adults, with effect sizes that implicate a practical significance of the effects. However, Breit et al. (2022) also showed that age differentiation in children and adolescents was not supported. Instead, small but negligible effect sizes were found for age dedifferentiation in adolescents.

The LSEM method has been extended to two moderator variables by Hartung et al. (2018). Molenaar (2021) proposed a semiparametric moderated factor modeling approach in which no assumption concerning the functional form between the moderator and the model parameters are imposed. In contrast to the original definition of LSEM (Hildebrandt et al. 2009), some model parameters are allowed to be invariant across the continuous moderator variable.

LSEM is closely related to moderated nonlinear factor analysis (MNFA; Bauer 2017; Curran et al. 2014; Molenaar and Dolan 2012). In MNFA, a functional form of SEM model parameters as a function of a single moderator (or multiple moderators) is imposed. In this sense, MNFA is often more confirmatory than LSEM. Nevertheless, differentiation hypotheses were also investigated by means of MNFA (Molenaar et al. (2010a, 2010b, 2011, 2017)). A tutorial on how to apply MNFA using the R package OpenMx (Boker et al. 2011) was given by Kolbe et al. (2022). LSEM also bears a similarity to the approach of individual parameter change (Oberski 2013; Arnold et al. (2020, 2021). Variation in SEM model parameters can also be tested with score-based invariance tests (Huth et al. 2022; Merkle and Zeileis 2013; Wang et al. 2014).

LSEM has been implemented in the R package sirt (Robitzsch 2023b) as a wrapper to the popular SEM package lavaan (Rosseel 2012). Moreover, the R package umx (Bates et al. 2019) can also be utilized for LSEM estimation.

This article reviews and extends LSEM estimation methods and discusses the implementation in the R package sirt. In previous literature, LSEM was fitted as a sequence of models that are separately evaluated as each value of the moderator variables. In this article, a joint estimation approach is proposed that is a simultaneous estimation method across all moderator values and also allows some model parameters to be invariant with respect to the moderator. Sufficient detail on the core estimation functions in the sirt package is provided. The article also evaluates two significance testing approaches to assess whether the moderator values are related to a model parameter in two simulation studies. Finally, an empirical example demonstrates the usefulness of the LSEM methodology.

The remainder of this article is structured as follows. Section 2 overviews the most important LSEM applications in the literature. In Section 3, different LSEM estimation and significance testing approaches are presented. Details about LSEM implementation in the sirt package can be found in Section 4. Section 5 discusses R input code and R output of an LSEM analysis involving illustrative datasets. Section 6 includes a simulation study investigating parameter recovery in LSEM regarding bias and root mean square error. Section 7 includes a simulation study that investigates different estimators of variability in parameter curves and the statistical properties of significance tests of parameter variation. In Section 8, an empirical example is presented that reanalyzes SON-R intelligence data for children aged between 212 and 7 years. Finally, Section 9 closes with a discussion.

## 2. Review of LSEM Applications

We now review important LSEM applications to demonstrate that this method is widely applied in substantive research. The original LSEM publication of Hildebrandt et al. (2009) (“Complementary and competing factor analytic approaches for the investigation of measurement invariance”) has been cited 93 times and 80 times, according to Google Scholar and ResearchGate (accessed on 18 July 2023), respectively. The second methodological LSEM publication by Hildebrandt et al. (2016) (“Exploring factor model parameters across continuous variables with local structural equation models”) has been cited 111 times, 89 times, and 77 times, according to Google Scholar, ResearchGate, and Web of Science (accessed on 18 July 2023), respectively. Hence, one could say that LSEM fills some niche in the researcher’s methodological toolbox.

In the following, some LSEM applications are briefly described. The studies are loosely organized according to the fields of application.

Olaru and Allemand (2022) examined differential and correlated change in personality across the adult lifespan using LSEM. Brandt et al. (2022) applied LSEM to four waves of data obtained with the full NEO Personality Inventory collected over 11 years from 1667 adults in a US sample using age as a continuous moderator. Hartung et al. (2021) investigated the age-moderated covariance structure of the satisfaction with life scale (SWLS) and the domains of health satisfaction and financial satisfaction using LSEM. Olaru et al. (2019) analyzed NEO personality indicators across ages between 16 and 66 years by means of LSEM. They selected items for short scales that had the greatest extent of measurement invariance across age. Seifert et al. (2022) studied whether the rank-order stability of personality increases until midlife and declines later in old age and found that this inverted U-shaped pattern was not consistently observed in two reanalyzes utilizing LSEM. Loneliness across different age levels was investigated by LSEM in Entringer and Gosling (2022) and Panayiotou et al. (2022). Van den Akker et al. (2021) applied LSEM for students aged between 8 and 18 years to investigate whether levels of conscientiousness and agreeableness decrease when levels of neuroticism increase, indicating a dip in personality maturation. Gnambs (2013) applied LSEM in a multitrait multi-informant meta-analysis for the big five factors.

Hartung et al. (2022) investigated the structure of the “dark personality factor” across age and gender using LSEM. Krasko and Kaiser (2023) investigated measurement invariance across age for the dark triad by means of LSEM.

Bratt et al. (2018) investigated levels of perceived age discrimination across early to late adulthood by employing LSEM, using data from the European social survey (ESS) collected in 29 countries. Dutton and Kirkegaard (2022) applied LSEM to investigate a particular question about the association between religiousness and intelligence. Allemand et al. (2022) used LSEM to investigate the effects of continuous age and COVID-19 virus worry on mean levels and correlations between gratitude and remaining opportunities and time. Allemand et al. (2021) examined age-related psychometrics and differences in the measurement, mean-levels, variances, and correlations of gratitude and future time perspective across adulthood using data in a representative Swiss sample for participants aged between 19 and 98 years.

Schroeders et al. (2015) studied the differentiation fluid and crystallized intelligence in German students of grades 5 to 12. Watrin et al. (2022) studied the age differentiation hypothesis of declarative knowledge, as proposed in Cattell’s investment theory. Hülür et al. (2011) studied with LSEM whether cognitive abilities become more differentiated with increasing age during childhood for children from age 2.5 to 7. Hartung et al. (2020) tested whether associations among executive functions strengthened from middle childhood to adolescence using cross-sectional data from a sample of children aged between 7 and 15 years. Gnambs and Schroeders (2020) examined the effects of cognitive abilities on the factor structure of the Rosenberg self-esteem scale across age by means of LSEM. Whitley et al. (2016) explored cross-sectional associations of age with five cognitive tests (word recall, verbal fluency, subtraction, number sequence, and numerical problem solving) in a large representative sample aged between 16 and 100 living in the UK. Breit et al. (2020) investigated ability differentiation, developmental differentiation, and their interaction with LSEM in two studies. Breit et al. (2021) provided a review of the literature on ability and developmental differentiation effects in children and youths. Breit et al. (2023) studied ability differentiation, including creativity measures, through LSEM for German students aged between 12 and 16 years.

Hildebrandt et al. (2010) employed LSEM to investigate structural invariance and age-related performance differences in face cognition. Hildebrandt et al. (2013) studied the specificity of face cognition compared with object cognition from individual differences and aging perspective by determining the amount of overlap between these abilities at the level of latent constructs across age. By utilizing LSEM, Liu et al. (2022) found that individual differences in white matter microstructure of the face processing brain network were more differentiated from global fibers with increasing ability.

LSEM was also applied in behavioral neurosciences Kaltwasser et al. (2017). Jokić-Begić et al. (2019) used LSEM for assessing measurement invariance across age for cyperchondria, a process of increased anxiety over one’s health as a result of excessive online searching. Lodi-Smith et al. (2021) found that autism characteristics measured by the autism-spectrum quotient scale were not strongly associated with age by utilizing LSEM. Cox et al. (2016) used LSEM to quantify microstructural properties of the human brain’s connections for understanding normal ageing and disease (see also Briley et al. (2015b)). Researchers de Mooij et al. (2018) used LSEM to study differences within and between brain and cognition across the adult life span. Zheng et al. (2019) investigated whether genetic and environmental influences on achievement goal orientations shift were moderated with age. Madole et al. (2019) applied LSEM in network analysis as a method for investigating symptom-level associations that underlie comorbidity connecting diagnostic syndromes.

Olaru et al. (2019) utilized LSEM in combination with ant colony optimization (see also Olaru and Jankowsky 2022) to resample and weight subjects to study differences in the measurement model across age as a continuous moderator variable.

An overview of different modeling strategies of LSEM for longitudinal data is presented in Olaru et al. (2020). Wagner et al. (2019) investigated through LSEM whether personality becomes more stable with age. They disentangled state and trait effects for the big five across the life span by applying LSEM to trait-state-occasion models. Gana et al. (2023) applied trait-state-occasion models in tandem with LSEM to investigate whether the characteristics of the depression EURO-D scale were associated with age.

LSEM was also applied to moderator variables different from age. Klieme and Schmidt-Borcherding (2023) employed LSEM to explore whether there is noninvariance for indicators of research self-efficacy regarding different training levels of students operationalized as the number of studied semesters. Weiss et al. (2020) investigated the threshold hypothesis of creativity by handling intelligence as a continuous moderator in LSEM. Schroeders and Jansen (2022) studied by means of LSEM whether the multidimensional structure of the science self-concept is moderated by levels of the cognitive ability in science. Basarkod et al. (2023) investigated whether reading self-concept dimensions vary across reading achievement levels in the PISA study. Olaru et al. (2022) examined the effects of family background on children’s receptive vocabulary using LSEM with latent growth curve models. Bolsinova and Molenaar (2019) (see also Bolsinova and Molenaar 2018) used LSEM for indicator-specific covariates and extended LSEM to the study of cognitive tests involving reaction times.

## 3. Estimating and Testing Local Structural Equation Models

### 3.1. Single-Group Structural Equation Model

In SEM, a measurement model is imposed that relates the observed variables X to latent variables η

(1) X=ν+Λη+ϵ.

In addition, the covariance matrix of ϵ is denoted by V; that is, Var(ϵ)=Ψ. Moreover, η and ϵ are multivariate normally distributed random variables. In addition, η and ϵ are assumed to be uncorrelated. In CFA, the multivariate normal (MVN) distribution is represented as η∼MVN(α,Φ) and ϵ∼MVN(0,Ψ). As we are only concerned with the covariance structure in SEM in this paper, we assume α=0 and E(X)=ν. Then, the covariance matrix of X in CFA can be computed as:

(2) $$\mathrm{Var}\left(X\right)=\Sigma \left(\theta \right)=\Lambda \Phi \Lambda^{⊤}+\Psi \;.$$

The parameter vector θ contains parameters in Λ, Φ, and Ψ that are estimated. Typically, the covariance matrix Σ is a constrained matrix determined by the specification (2).

In a general SEM, relationships among the latent variables η are modeled in path models. A matrix B of regression coefficients is specified such that:

(3) η=Bη+ζ,

where η denotes an endogeneous and ζ an exogeneous multivariate normally distributed latent variables. Note that (3) can be written as:

(4) $$\eta ={\left(I-B\right)}^{-1}\zeta \;,$$

where I denotes the identity matrix. In this case, the covariance matrix of X are represented in SEM as:

(5) $$\mathrm{Var}\left(X\right)=\Sigma \left(\theta \right)=\Lambda {\left(I-B\right)}^{-1}\Phi {\left[{\left(I-B\right)}^{-1}\right]}^{⊤}\Lambda^{⊤}+\Psi \;.$$

Some identification constraints must be imposed when estimating the covariance structure of the SEM in (2) or (5) (Bollen 1989; Bollen and Davis 2009). The purpose of identifying constraints primarily lies in a convenient interpretation of latent variables η and is not primarily driven by improving the efficiency of estimating Σ.

When modeling multivariate normally distributed data without missing data, the empirical covariance matrix S is a sufficient statistic for the unknown covariance matrix Σ. Hence, S is also sufficient for the parameter vector θ of the SEM in (2) or (5).

### 3.2. Multiple-Group Structural Equation Model

We now describe the general estimation of a multiple-group SEM. There exist *G* known groups g=1,…,G. The allocation of a group to a subject is known in this case. Assume that group *g* has $N_{g}$ subjects and an empirical covariance matrix $S_{g}$. The population covariance matrices are denoted by $\Sigma_{g}$ (g=1,…,G). The model-implied covariance matrices are denoted by $\Sigma_{g}\left(\theta \right)$(g=1,…,G). The unknown parameter vector θ can have common parameters across groups and parameters that are group-specific. For example, in a CFA, equal factor loadings and item intercepts across groups are frequently imposed (i.e., measurement invariance holds; Meredith 1993; Putnick and Bornstein 2016) by assuming the same loading matrix Λ across groups, while covariance matrices of latent variables or the matrix B of regression coefficients are allowed to differ across groups.

Up to constants, the maximum likelihood (ML) fitting function of the unknown parameter θ for the covariance structure in the multiple-group SEM is given by (see Bollen 1989 and Jöreskog et al. 2016):

(6) $$F\left(\theta ;{\left\{S_{g}\right\}}_{g}\right)=\sum_{g=1}^{G}N_{g}\left(\log|\Sigma_{g}\left(\theta \right)|+\mathrm{tr}\left(S_{g}\Sigma_{g}{\left(\theta \right)}^{-1}\right)-\log\left|S_{g}\right|-I\right)\;.$$

Note that *I* refers to the number of observed variables; that is, the dimension of X. The set ${\left\{S_{g}\right\}}_{g}$ denotes the set of *G* empirical covariance matrices that are sufficient statistics in multiple-group SEM estimation. The parameter vector θ is estimated by minimizing *F* in (6) and is denoted as the ML estimate. The estimated parameter is denoted by $\hat{\theta }$.

In practice, the model-implied covariance matrix can be misspecified (Boos and Stefanski 2013; Gourieroux et al. 1984; Kolenikov 2011; White 1982), and θ is a pseudo-true parameter defined as the minimizer of the fitting function *F* in (6). Importantly, θ does not refer to a parameter of the data-generating model in this case. In contrast, it should be interpreted as a summary of the data that are of central interest to the researcher.

The ML fitting function (6) can be considered a special case of discrepancy function. To this end, we define a general discrepancy function D(S,Σ) between an empirical covariance matrix S and a population covariance matrix Σ. The real-valued nonnegative function D should only attain the value zero if S=Σ (i.e., for correctly specified models). For the ML fitting function, the discrepancy function D is defined as:

(7) $$D\left(S,\Sigma (\theta )\right)=\log|\Sigma (\theta )|+\mathrm{tr}(S\Sigma {\left(\theta \right)}^{-1})-\log|S|-I\;.$$

Using definition (7), we can rewrite (6) as:

(8) $$F\left(\theta ;{\left\{S_{g}\right\}}_{g}\right)=\sum_{g=1}^{G}N_{g}D\left(S_{g},\Sigma_{g}\left(\theta \right)\right)\;,$$

and $\hat{\theta }$ is the minimizer of $F(\theta ;{\left\{S_{g}\right\}}_{g})$.

If an age moderator variable *A* is available, an SEM can, in principle, be estimated for all subgroups of subjects for different values of the age variable. In practice, sample sizes for concrete age values might be too small for separate estimation of the SEM. Moreover, discretizing the values of a continuous moderator variable *A* into *G* distinct groups of subjects might not be preferred due to loss of information (Hildebrandt et al. 2009). To circumvent these issues, LSEM has been proposed. We discuss LSEM estimation methods in the next subsections.

### 3.3. Local Weighting

Instead of grouping subjects that fall within a given range of the moderator, as in multiple-group SEMs, observations are locally weighted around focal points (i.e., specific values of the continuous moderator variable) in LSEM. In previous studies, SEMs are sequentially estimated on the basis of weighted samples of observations at all focal points (i.e., the pointwise LSEM estimation approach, see Section 3.5).

In LSEM, researchers are interested in investigating moderator-specific covariance structures. That is, they aim to model conditional covariances:

(9) Var(X|A=a)=Σ(a)

As argued in the previous section, sample sizes might be too small for estimating Σ(a) only for subjects with A=a. To this end, subjects with moderator values *a* sufficiently close to a focal point $a_{t}$ (i.e., a chosen value of the moderator variable *A*) should also enter the estimation. For each focal point $a_{t}$ and each subject *n*, weights $w_{nt}$ are computed that reflect the distance of the moderator value (e.g., a value of age) of person *n* (i.e., $a_{n}$) and the focal point $a_{t}$. If $a_{n}=a_{t}$, the weight should be one, and it should be zero for age values $a_{n}$ that strongly differ from $a_{t}$.

The computation of weights relies on a kernel function *K* that is chosen by the researcher (Hildebrandt et al. (2009, 2016). The real-valued kernel function fulfills the properties K(0)=1, K(x)=K(−x) (i.e., it is a symmetry function), K(x)≥0 for all x∈R, and *K* is a decreasing function for x≥0. The subject-specific weight $w_{nt}$ for subject *n* at a focal point $a_{t}$ with a pre-specified bandwidth bw is computed as:

(10) $$w_{nt}=K\left(\frac{a_{n}-a_{t}}{bw}\right)\;.$$

By the definition of *K*, weights are bounded within the interval [0,1].

Typical choices of the weight function in the literature of nonparametric regression or density estimation are the Gaussian kernel, the Epanechnikov kernel, and the uniform kernel function. The Gaussian kernel function is defined as:

(11) $$K\left(x\right)=\exp\left(-x^{2}/2\right)\;.$$

In density estimation involving the Gaussian kernel function, an optimal bandwidth is given by $bw=hN^{-1/5}\sigma_{A}$ with h=1.1, and $\sigma_{A}$ is the standard deviation of the age moderator variable (Silverman 1986). The parameter *h* is referred to as the bandwidth factor in this article. The Epanechnikov kernel function is defined as:

(12) $$K\left(x\right)=\left\{\begin{array}{cc} \frac{3}{4}\left(1-x^{2}\right) & \mathrm{for}\;|x|\leq 1\\ 0 & \mathrm{for}\;|x|>1 \end{array}\right.\;.$$

For age values $a_{n}$ with $|a_{n}-a_{t}|>bw$, weights $w_{nt}$ are zero. Finally, the uniform kernel function is defined as:

(13) $$K\left(x\right)=\left\{\begin{array}{cc} 1 & \mathrm{for}\;|x|\leq 1\\ 0 & \mathrm{for}\;|x|>1 \end{array}\right.\;.$$

The uniform kernel can be used to define weights so that they reflect the discretization of the continuous age variable *A* into *G* distinct groups. The estimated LSEM will provide parameter results that are identical to the multiple-group SEM if the same identification constraints are utilized.

### 3.4. Estimation of Conditional Means and Conditional Covariances

We now describe the estimation conditional covariances Σ(a). In a practical implementation of the LSEM, researchers define a discrete grid of moderator values $a_{1},a_{2},\ldots ,a_{T}$ (i.e., the focal points) of the age variable *A*. In most applications, a grid of equidistant focal points is chosen (Hildebrandt et al. 2016). However, the grid of focal points could also be chosen in such a way that it mimics the empirical distribution of the moderator variable. For example, researchers might use empirical percentiles of the moderator variable (e.g., a grid of 10 focal points using the *p*th percentile for p=5,15,…,95).

To estimate conditional covariances at a focal point $a_{t}$, we first compute the conditional mean function E(X|A=a) for $X=(X_{1},\ldots ,X_{I})$. For a variable $X_{i}$ for i=1,…,I, a local quadratic regression model is specified to estimate the conditional mean at focal point $a_{t}$. That is, one minimizes:

(14) $$\left({\hat{\gamma }}_{it0},{\hat{\gamma }}_{it1},{\hat{\gamma }}_{it2}\right)=\underset{\left(\gamma_{it0},\gamma_{it1},\gamma_{it2}\right)}{arg min}\left\{\sum_{n=1}^{N}w_{nt}{\left(x_{in}-\gamma_{it0}-\gamma_{it1}\left(a_{nt}-a_{t}\right)-\gamma_{it2}{\left(a_{nt}-a_{t}\right)}^{2}\right)}^{2}\right\}$$

The conditional mean estimate of $\mu_{i}\left(a_{t}\right)=E\left(X_{i}|A=a_{t}\right)$ is given by ${\hat{\mu }}_{i}\left(a_{t}\right)={\hat{\gamma }}_{it0}$. Note that the minimization in (14) is a weighted least squares estimation problem for a linear regression (i.e., it is linear in model parameters) and closed formulae are available for estimating $\left(\gamma_{it0},\gamma_{it1},\gamma_{it2}\right)$ (see Fox 2016).

We now describe the estimation of conditional covariances $\sigma_{ij}\left(a_{t}\right)=\mathrm{Cov}\left(X_{i},X_{j}|A=a_{t}\right)$. First, residuals $e_{nit}$ are computed using local quadratic regression parameters defined in (14) as:

(15) $$e_{nit}=x_{in}-{\hat{\gamma }}_{it0}-{\hat{\gamma }}_{it1}\left(a_{nt}-a_{t}\right)-{\hat{\gamma }}_{it2}{\left(a_{nt}-a_{t}\right)}^{2}\;.$$

The estimate of the conditional covariances $\sigma_{ij}\left(a_{t}\right)$ can be obtained by simple weighting or a local regression model.

In the weighting approach, one estimates:

(16) $${\hat{\sigma }}_{ij}\left(a_{t}\right)=W_{t}^{-1}\sum_{n=1}^{N}w_{nt}e_{nit}e_{njt}\;,$$

where $W_{t}=\sum_{n=1}^{N}w_{nt}$. This approach was advocated in Hildebrandt et al. (2009) and Hildebrandt et al. (2016).

In recently proposed local regression modeling (see Olaru et al. 2020), one also specifies a local quadratic regression estimation problem for the computation of the conditional covariance:

(17) $$\left({\hat{\delta }}_{ijt0},{\hat{\delta }}_{ijt1},{\hat{\delta }}_{ijt2}\right)=\underset{\left(\delta_{ijt0},\delta_{ijt1},\delta_{ijt2}\right)}{arg min}\left\{\sum_{n=1}^{N}w_{nt}{\left(e_{nit}e_{njt}-\delta_{ijt0}-\delta_{ijt1}\left(a_{nt}-a_{t}\right)-\delta_{ijt2}{\left(a_{nt}-a_{t}\right)}^{2}\right)}^{2}\right\}\;.$$

The estimate of the conditional covariance is given as ${\hat{\sigma }}_{ij}\left(a_{t}\right)={\hat{\delta }}_{ijt0}$.

Note that the estimation of the conditional mean function in (14) and the conditional covariance function in (17) is essentially equivalent, except for the case that the former uses the values $x_{ni}$ as the dependent variable $x_{ni}$ (i.e., indicator *i*), while the latter uses the product residual $e_{nit}e_{njt}$ of variables for indicators *i* and *j* for the computation of the moderator-specific conditional covariance.

The steps can be repeated for all pairs of variables *i* and *j* (i,j=1,…,I) and all focal points $a_{t}$ (t=1,…,T). The resulting estimated conditional covariance matrices at focal points $a_{t}$ are denoted by ${\hat{\Sigma }}_{t}$ (t=1,…,T). The estimated covariance matrices ${\hat{\Sigma }}_{t}$ are not guaranteed to be positive definite. Therefore, the estimate might be slightly modified to determine a close matrix to ${\hat{\Sigma }}_{t}$ that fulfills the positive definiteness property (Bentler and Yuan 2011).

LSEM estimation methods rely on the estimated conditional covariances. Three different estimation approaches are described in Section 3.5, Section 3.6, and Section 3.8.

### 3.5. Pointwise LSEM Estimation

Pointwise LSEM estimation relies on the idea that a separate SEM is fitted to each focal point $a_{t}$. The resulting parameter estimates ${\hat{\theta }}_{t}$ are plotted or analyzed as a function of the age variable *A*. More formally, based on the conditional covariance estimate ${\hat{\Sigma }}_{t}$, at each focal point $a_{t}$, the following fitting function is minimized:

(18) $$F\left(\theta_{t};{\hat{\Sigma }}_{t}\right)=D\left({\hat{\Sigma }}_{t},\Sigma_{t}\left(\theta_{t}\right)\right)\;,$$

where ${\hat{\theta }}_{t}$ denotes the minimizer of $F(\theta_{t};{\hat{\Sigma }}_{t})$. Note that in (18), the distance between the empirical conditional covariance ${\hat{\Sigma }}_{t}$ and the model-implied conditional covariance $\Sigma_{t}\left(\theta_{t}\right)$ at the focal point $a_{t}$ is minimized. This approach was proposed by Hildebrandt et al. (2009, 2016). The minimization in (18) is not restricted to ML estimation and can also be applied to weighted least estimation in SEM (Browne 1974) or model-robust fitting functions (Robitzsch 2023a).

Model fit statistics, such as RMSEA, SRMR, or TLI, are computed at each value of the focal point. Note that pointwise LSEM estimation provides parameter curves across different values of the moderator variable.

The pointwise LSEM estimation method allows the parameter vector θ(a) to vary freely across *a*. However, this flexibility sometimes hinders interpretation. Moreover, some researchers might prefer to impose invariance constraints for some of the model parameters (Leitgöb et al. 2023). For this reason, a joint LSEM estimation approach is proposed that is described in the next Section 3.6.

### 3.6. Joint LSEM Estimation with Invariance Constraints

While pointwise LSEM estimation tackles the estimation problem by successively and separately estimating an SEM at each of the focal points, joint LSEM estimation defines a single estimation function that involves conditional covariance matrices of all focal points. By doing so, the parameter vector θ can contain parameters that are specific to each focal point and parameters that do not vary for different values of age. The fitting function is defined as:

(19) $$F\left(\theta ;{\left\{{\hat{\Sigma }}_{t}\right\}}_{t}\right)=\sum_{t=1}^{T}W_{t}D\left({\hat{\Sigma }}_{t},\Sigma_{t}\left(\theta \right)\right)\;,$$

where $\hat{\theta }$ is the minimizer of $F(\theta ;{\left\{{\hat{\Sigma }}_{t}\right\}}_{t})$ and $W_{t}=\sum_{n=1}^{N}w_{nt}$ is the sum of weights specific to each focal point $a_{t}$. Note that (19) looks like a fitting function in multiple-group SEM estimation. However, subjects can enter multiple groups (i.e., focal points) because they enter the estimated conditional covariances multiple times according to the weights $w_{nt}$. Hence, the fitting function *F* in (19) will not be an ML fitting function and falls in the general class of M-estimation problems (Stefanski and Boos 2002).

The parameter vector θ can be decomposed into components $\theta =(\theta_{0},\theta_{1},\ldots ,\theta_{T})$, where $\theta_{0}$ contains parameters that are invariant across age, and $\theta_{t}$ for t≥1 contain the parameters that vary across age values. The fitting function in (19) can then be rewritten as:

(20) $$F\left(\theta_{0},\theta_{1},\ldots ,\theta_{T};{\left\{{\hat{\Sigma }}_{t}\right\}}_{t}\right)=\sum_{t=1}^{T}W_{t}D\left({\hat{\Sigma }}_{t},\Sigma_{t}\left(\theta_{0},\theta_{t}\right)\right)\;.$$

Note that the originally proposed pointwise estimation of the fitting function in (18) is equivalent to joint LSEM estimation in (20) if there does not exist invariant model parameters $\theta_{0}$.

In joint LSEM estimation, global model fit statistics are computed. These fit statistics can be interpreted similarly as in multiple-group SEMs.

### 3.7. Estimation of DIF Effects

In joint LSEM estimation defined by the fitting function *F* in (20), some parameters (i.e., the parameter vector $\theta_{0}$) have invariance constraints across the age moderator variable. These invariance constraints ease interpretation and have the advantage of specifying parsimonious SEMs. However, researchers might be interested in what would happen if these invariance constraints were freed.

Violations of measurement invariance are referred to as differential item functioning (DIF) in item response theory literature (Mellenbergh 1989; Holland and Wainer 1993; Millsap 2011). Noninvariant parameters are referred to as DIF effects in this literature. We also use this notation and now discuss the estimation of DIF effects. DIF effects emerge if all estimated age-specific parameters ${\hat{\theta }}_{t}$ (t≥1) are held fixed in (20), and the entries of the parameter vector $\theta_{0}$ are allowed to vary across age. We denote the focal-point-specific estimates of DIF effects by $\delta_{t}$. To this end, invariant parameters $\theta_{0}$ are replaced with $\delta_{1},\ldots ,\delta_{T}$, and the following fitting function *F* is minimized to obtain DIF effect estimates ${\hat{\delta }}_{t}$ (t=1,…,T):

(21) $$F\left(\delta_{1},\ldots ,\delta_{T},{\hat{\theta }}_{1},\ldots ,{\hat{\theta }}_{T};{\left\{{\hat{\Sigma }}_{t}\right\}}_{t}\right)=\sum_{t=1}^{T}W_{t}D\left({\hat{\Sigma }}_{t},\Sigma_{t}\left(\delta_{t},{\hat{\theta }}_{t}\right)\right)\;.$$

Note that there are no invariant model parameters in (21), and the DIF effects $\delta_{t}$ at the focal point $a_{t}$ could alternatively be obtained by pointwise minimization of:

(22) $$F\left(\delta_{t},{\hat{\theta }}_{t};{\hat{\Sigma }}_{t}\right)=D\left({\hat{\Sigma }}_{t},\Sigma_{t}\left(\delta_{t},{\hat{\theta }}_{t}\right)\right)\;.$$

The estimated DIF effects can be plotted or analyzed as a function of the age moderator to investigate whether the invariance constraints are substantially violated.

### 3.8. Joint LSEM Estimation with More General Parameter Constraints and Relation to Moderated Nonlinear Factor Analysis

In this subsection, joint LSEM estimation is slightly generalized. The fitting function is the same as in (20), but constrains across focal-point-specific parameters $\theta_{t}$ are allowed. In particular, we discuss the implementation of linear, quadratic, and piecewise linear or quadratic parameter constraints.

Assume a parameter curve $\theta (a_{t})$ for a particular parameter. Furthermore, assume that the focal points are equidistant; that is, $a_{t+1}-a_{t}=\Delta$ are equal for t=1,…,T−1.

We first describe a linear parameter constraint. A linear function of a parameter θ for age values *a* is given by $f\left(a\right)=\alpha_{0}+\alpha_{1}a$. The first derivative of *f* is constant, and it holds that $f^{\prime }\left(a_{t+1}\right)=f^{\prime }\left(a_{t}\right)=\alpha_{1}$. Hence, the equality in derivatives can be translated into equalities in first-order differences in model parameters:

(23) $$\theta \left(a_{t+2}\right)-\theta \left(a_{t+1}\right)=\theta \left(a_{t+1}\right)-\theta \left(a_{t}\right)\;.$$

These constraints can be added in multiple-group SEM in typical SEM software such as lavaan (Rosseel 2012).

A quadratic function of a parameter is given by $f\left(a\right)=\alpha_{0}+\alpha_{1}a+\alpha_{2}a^{2}$. This function has constant second-order derivatives; that is, $f^{\prime \prime }\left(a_{t+1}\right)=f^{\prime \prime }\left(a_{t}\right)=2\alpha_{2}$. Hence, second-order differences in parameter values are constant, which translates into:

(24) $$\theta \left(a_{t+3}\right)-2\theta \left(a_{t+2}\right)+\theta \left(a_{t+1}\right)=\theta \left(a_{t+2}\right)-2\theta \left(a_{t+1}\right)+\theta \left(a_{t}\right)\;.$$

Similarly, cubic parameter constrains can be implemented by recognizing that the third-order differences in parameter values are constant. A slightly more tedious constraint than (24) can be derived.

The linearity and quadratic constraints in (23) and (24) can also be applied if parameter curves are broken into segments. Hence, piecewise linear or quadratic functions can be applied.

Applying (piecewise) quadratic parameter functions in joint LSEM estimation can be interpreted as a kind of smoothing procedure to stabilize parameter estimation. Furthermore, the raw data are smoothed when computing the estimated conditional covariance matrices ${\hat{\Sigma }}_{t}$. Hence, researchers have two choices for how stabilizing parameter estimation in LSEM.

Notably, parameter constraints in joint LSEM estimation are estimates of MNFA in a particular case. If the age moderator values *A* has only values at the grid of equidistant focal points $a_{1},\ldots ,a_{T}$, then using the uniform kernel with $bw=(a_{2}-a_{1})/2$ is equivalent to MNFA with appropriate parameter constraints. Such an approach is described in Tucker-Drob (2009).

### 3.9. Parameter Curve Summaries and Significance Testing

Finally, we discuss the definition of summary statistics and the test of significant parameter variation across age. Let $\theta (a_{t})$ be a parameter curve of some model parameter estimated at focal points $a_{t}$ (t=1,…,T). The parameter curve θ(a) can be summarized by the mean and the standard deviation. Let $f(a_{t})$ be the discrete density of the age variable *A* at focal point $a_{t}$ and assume that $\sum_{t=1}^{T}f\left(a_{t}\right)=1$. The (weighted) average value of the parameter curve (i.e., the mean) is given as:

(25) $$M_{\theta (a)}=\sum_{t=1}^{T}f\left(a_{t}\right)\theta \left(a_{t}\right)\;.$$

In practice, an estimate of (25) is obtained by

(26) $${\hat{M}}_{\theta (a)}=\sum_{t=1}^{T}\hat{f}\left(a_{t}\right)\hat{\theta }\left(a_{t}\right)\;.$$

The standard deviation of a parameter curve quantifies the variability of a parameter curve across age and is given by:

(27) $${\mathrm{SD}}_{\theta (a)}=\sqrt{\sum_{t=1}^{T}f\left(a_{t}\right){\left(\theta \left(a_{t}\right)-M_{\theta (a)}\right)}^{2}}\;.$$

An estimate of the standard deviation defined in (27) is given by:

(28) $${\hat{\mathrm{SD}}}_{\theta (a)}=\sqrt{\sum_{t=1}^{T}\hat{f}\left(a_{t}\right){\left(\hat{\theta }\left(a_{t}\right)-{\hat{M}}_{\theta (a)}\right)}^{2}}\;.$$

The sample estimate ${\hat{\mathrm{SD}}}_{\theta (a)}$ is always positive in finite samples if no invariance constraints are imposed. Hence, the naive standard deviation estimate in (28) will be positively biased. The bootstrap resampling procedure (Efron and Tibshirani 1994) can be used to reduce the bias in an estimate of ${\mathrm{SD}}_{\theta (a)}$. For LSEM, nonparametric bootstrap is implemented, which resamples subjects with replacement. The pointwise standard deviation of a parameter value across bootstrap samples can be used as a standard error estimate. A bias-corrected estimate of the standard deviation is obtained by:

(29) $${\hat{\mathrm{SD}}}_{\theta (a),\mathrm{bc}}={\mathrm{sqrt}}_{+}\left({\hat{\mathrm{SD}}}_{\theta (a)}^{2}-B_{\theta (a)}\right)\;,$$

where ${\mathrm{sqrt}}_{+}\left(x\right)=\sqrt{\max(x,0)}$ and $B_{\theta (a)}$ is the finite-sample bias of ${\hat{\mathrm{SD}}}_{\theta (a)}^{2}$ that can be determined by bootstrap resampling (Efron and Tibshirani 1994). A t-statistic for significant variation in an estimated parameter curve can be computed as:

(30) $$t={\hat{\mathrm{SD}}}_{\theta (a),\mathrm{bc}}/SE\;,$$

where SE is the standard deviation of ${\hat{\mathrm{SD}}}_{\theta (a)}$ values defined in (28) across different bootstrap samples. Note that this test procedure relies on a normal distribution assumption for the test statistic *t*, although it is probably an incorrect null distribution.

An alternative test for parameter variation is based on a Wald test. A covariance matrix estimate V for the vector $\xi =(\theta \left(a_{1}\right),\ldots ,\theta \left(a_{T}\right))$ can be obtained from bootstrap. It is assumed that $\hat{\xi }$ is multivariate normally distributed. Let H be a (T−1)×T matrix that implements equality constraints across the values of the parameter curve. The null hypothesis of no parameter variation is given by Hξ=0. Consider the Wald test statistic:

(31) $$\chi^{2}={\hat{\xi }}^{⊤}H^{⊤}{\left(H^{⊤}VH\right)}^{-1}H\hat{\xi }$$

This statistic is chi-square distributed with T−1 degrees of freedom.

In previous work, a permutation test has been proposed for testing parameter variation (Hartung et al. 2022; Hildebrandt et al. (2009, 2016)). A permutation test simultaneously assesses the effects on all parameters. In contrast, the test based on the standard deviation (30) and the Wald test (31) relies on a fitted model without modifying all other model parameters. Hence, we tend to favor the latter statistics over the permutation test.

## 4. Implementation of Local Structural Equation Models in the Sirt Package

In this section, we discuss the implementation of LSEM in the R (R Core Team 2023) package sirt (Robitzsch 2023b). The CRAN version can be installed within R using utils::install.packages(’sirt’), while the most recent GitHub version can be installed employing devtools::install_github(’alexanderrobitzsch/sirt’). The four primary LSEM functions are sirt::lsem.estimate(), sirt::lsem.bootstrap(), sirt::lsem.test() and sirt::lsem.permutationTest(), which will be discussed below. The new CRAN release of sirt from August 2023 (sirt 3.13-228; https://cran.r-project.org/web/packages/sirt/ accessed on 11 August 2023) includes the functionality described in this article.

LSEM estimation in sirt provides a wrapper to the SEM package lavaan (Rosseel 2012). The model specification follows the lavaan syntax, which eases the familiarity with R code for LSEM estimation because lavaan seems to be the most popular open-source SEM software.

In Listing 1, the main function sirt::lsem.estimate() is displayed. This function is the main LSEM estimation function. We now discuss the most important arguments in detail.

**Listing 1.** LSEM function sirt::lsem.estimate().

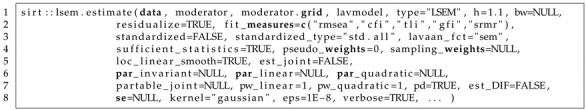



In data, a data frame must be provided by the user. The data frame should also include the moderator variable, whose variable name must be specified in moderator. The set of focal points can be defined as a vector moderator.grid. In lavmodel, lavaan syntax must be provided for estimating the LSEM. The default of the argument type is “LSEM”; that is, an LSEM is estimated. By choosing type=”MGM”, a multiple-group model with a discretized moderator variable is estimated. The bandwidth in sirt::lsem.estimate() can be specified by h or bw. The arguments are related through the formula:

(32) $$bw=hN^{-1/5}{\hat{\sigma }}_{A}\;,$$

where ${\hat{\sigma }}_{A}$ denotes the estimated standard deviation of the moderator variable *A* (i.e., the argument moderator). The logical argument residualize indicates whether local regression smoothing of the mean structure should be applied before estimating conditional covariances. The argument fit_measures defines fit statistics available in lavaan that should be included in the LSEM output. The logical argument standardized defines whether standardized parameters should appear in the LSEM output. The type of standardization is specified in standardized_type whose conventions follow the lavaan package. In lavaan_fct, the lavaan function is specified that is used for LSEM estimation. The default lavaan_fct="sem" refers to lavaan::sem(). Other options are "cfa" (for lavaan::cfa()) and "lavaan" (for lavaan::lavaan()). The logical argument sufficient_statistics indicates whether sufficient statistics (i.e., conditional mean and conditional covariances) should be used in estimation. Without missing data, ML can always rely on sufficient statistics. However, in the presence of missing data, conditional covariance matrices are estimated based on pairwise deletion. However, if full information maximum likelihood was utilized, the mean structure cannot be properly residualized. Hence, researchers are advised either to believe in missing data mechanisms close to missing completely at random that justify the usage of pairwise deletion or to apply an appropriate multiple imputation procedure prior to LSEM analysis if there are missing values in the dataset.

Users can also input a vector of sampling weights in sampling_weights. The logical argument loc_linear_smooth defines whether local quadratic regression (see (17)) should be applied in the estimation of conditional covariances. If the default loc_linear_smooth=TRUE is changed into loc_linear_smooth=FALSE, the weighting formula (16) is utilized. The logical argument est_joint indicates whether joint LSEM estimation (i.e., the default; see Section 3.6 or Section 3.8) or pairwise LSEM estimation (see Section 3.5) is applied. Invariant model parameters can be specified in the vector argument par_invariant. If there are some invariant parameters, joint LSEM estimation is automatically chosen (i.e., est_joint=TRUE). Linear or quadratic parameter constraints on model parameters (see Section 3.8) can be specified with par_linear and par_quadratic, respectively. The number of segments in piecewise linear or piecewise quadratic parameter constrained estimation can be specified with pw_linear or pw_quadratic. The default is that the constrains should be applied across all moderator values (i.e., there is only one segment of a piecewise linear or quadratic function). The argument partable_joint allows the input of a lavaan parameter table in joint estimation. This argument has the advantage that arbitrary parameter constraints can be specified by the user (e.g., additional equality constraints in piecewise quadratic functions). The logical argument pd indicates whether non-positive definite conditional covariance matrices should be smoothed to ensure positive definiteness. The logical argument est_DIF defines whether DIF effects should be estimated (see Section 3.7). Note that DIF effects can only be estimated if the LSEM model contains some invariant model parameters. The argument kernel allows the choice of the kernel function. Possible options are “gaussian”, “epanechnikov”, and “uniform”. Finally, the logical argument verbose indicates whether some output should be displayed in the R console when estimating the LSEM model.

Listing 2 displays the LSEM bootstrapping function in the sirt package. An object object must be provided that is the output of the sirt::lsem.estimate() function. The number of bootstrap samples can be specified by the argument R. Bootstrap can also be applied at the level of higher-order units. For example, school classes, schools, or organizations can be bootstrapped instead of bootstrapping subjects. Such a kind of cluster bootstrap is required if there is an additional dependency structure in the data. In this case, users can define a vector of cluster units in cluster. The sirt::lsem.bootstrap() also allows more general replication designs such as jackknife, balanced repeated replication, or half sampling (Kolenikov 2010) by providing an N×R matrix of resampling weights in the argument repl_design. In the case of more complex designs, a scale factor repl_factor must be defined by the user for a correct standard error computation. In the case of jackknife, it is 1 (or (R−1)/R), while it is 1/R in the case of bootstrap resampling. The bootstrap function sirt::lsem.bootstrap() is needed for computing the standard deviation statistic of parameter curves and its statistical inference (see Section 3.9). The sirt::lsem.bootstrap() function also allows an option for parallel computing. The number of employed cores can be specified by the argument n.core. The default is the use of one core which means that no parallel computing is applied in LSEM bootstrap estimation.

**Listing 2.** LSEM function sirt::lsem.bootstrap().

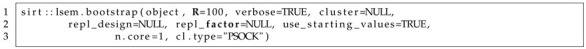



Listing 3 displays the LSEM function sirt::lsem.test() that performs the Wald tests for parameter variation (see Section 3.9). Instead of applying a test of the equality of a parameter curve on *T* focal points $a_{1},\ldots ,a_{T}$, the specification in models allows the test of significant regression parameters for a particular function. For example, a specification "FX=∼X1"=y∼m+I(m^2) tests whether the vector of the linear and the quadratic regression coefficient of the factor loading FX=∼X1 differs from (0,0). Note that sirt::lsem.test() requires the output of sirt::lsem.estimate() in mod and the output of the application of the bootstrap (or general resampling) of sirt::lsem.bootstrap() in bmod.

**Listing 3.** LSEM function sirt::lsem.test().

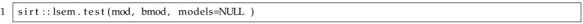



Listing 4 displays the LSEM function sirt::lsem.permutationTest() that carries out the permutation test for a statistical significance test for variation in parameter curves of the LSEM model Hildebrandt et al. (2009, 2016). In the permutation test, the values of the moderator variables are randomly resampled in the dataset to create a null distribution of parameter curves under the assumption of no relation to the moderator. The number of permutation samples can be specified in the argument B. As in sirt::lsem.bootstrap(), parallel computing can be requested by the number of cores in the argument n.core.

**Listing 4.** LSEM function sirt::lsem.permutationTest().

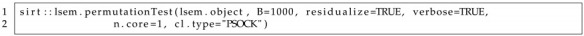



## 5. Illustrative Datasets

In this section, we illustrate LSEM estimation with the R package sirt. Three simulated datasets involving six variables X1, X2, X3, Y1, Y2, and Y3 are used for illustration. The analysis model is a two-dimensional factor model with a simple loading structure, where the first factor FX is measured by X1, X2, and X3, and the second factor FY is measured by Y1, Y2, and Y3. The moderator variable age was assessed at 13 time points, referring to ages 6,7,…,18. An anonymous reviewer pointed out that using 13 time points would look like longitudinal data. However, we only used the 13 time points for illustratory purposes. For example, there could be 13 cross-sectional age groups that are assessed.

The population parameters of the factor model for each age a=6,7,…,18 and each of the three datasets *DATA1*, *DATA2*, and *DATA3* can be found in the directory “*POPPARS*” at https://osf.io/puaz9/?view_only=63ffb2fd30f5400e89c59d03366bf793 (accessed on 3 June 2023). From these population parameters, 10,000 subjects were simulated at each of the 13 age points. The distribution at each age point exactly coincides with the specified conditional mean vector and the conditional covariance matrix (see, e.g., the lavaan::simulateData() function with the argument empirical=FALSE for a similar functionality). Data were simulated from a multivariate normal distribution. This simulation ensures that the population data involving 130,000 subjects (i.e., =13×10,000 subjects) exactly follows the specified covariance structure. In *DATA1*, all model parameters except for residual variances were assumed noninvariant. In *DATA2*, only the structural parameters (i.e., factor correlation and factor variances) were noninvariant, while factor loadings and residual variances were assumed invariant. In *DATA3*, all measurement and structural model parameters were assumed invariant. The population datasets and the data-generating model parameters can be found in the directory “*POPDATA*” at https://osf.io/puaz9/?view_only=63ffb2fd30f5400e89c59d03366bf793 (accessed on 3 June 2023). The illustrative datasets used in this section were subsamples of 2000 subjects from datasets *DATA1*, *DATA2*, and *DATA3*. The main motivation for using a subsample of the data is to show that LSEM produces some variability in model parameter estimates even if the model parameter is invariant across the moderator values in the data-generating model. The subsamples were created by random sampling without replacement from the population datasets. These datasets can be found in the directory “*ILLUSDATA*” at https://osf.io/puaz9/?view_only=63ffb2fd30f5400e89c59d03366bf793 (accessed on 3 June 2023).

Listing 5 contains the specification of the LSEM model involving two factors FX and FY. In lines 5–10 in Listing 5, the lavaan syntax for the factor model is specified in the string lavmodel. Line 13 in Listing 5 defines the parameter names (i.e., the factor loadings of X2, X3, Y2, and Y3) that are assumed invariant across the values of the moderator variable age. Line 16 in Listing 5 specifies the vector of focal points at which the LSEM model should be estimated. Lines 19–21 in Listing 5 contain the R command for applying sirt::lsem.estimate(). Note that the invariant model parameters are provided with the argument par_invariant, DIF effects were estimated due to est_DIF=TRUE, and the bandwidth factor *h* was chosen as 1.1. Joint LSEM estimation was applied because invariance constraints among parameters were imposed. In line 25 in Listing 5, the random seed is fixed, which ensures that bootstrap resampling will not change when applying code at a different time. Line 26 in Listing 5 specifies bootstrapping using sirt::lsem.bootstrap(). In total, R=200 bootstrap samples were utilized. Note that the specified factor model in Listing 5 is misspecified for the dataset *DATA1*, but correctly specified for the datasets *DATA2* and *DATA3*.

**Listing 5.** Illustrative datasets: Specification of LSEM with invariant factor loadings in sirt::lsem.estimate() and subsequent bootstrap in sirt::lsem.bootstrap().

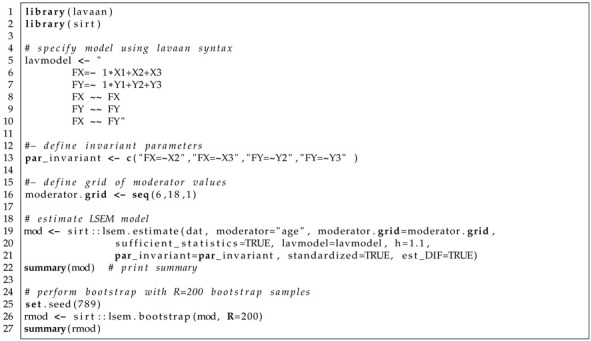



A part of R output of the sirt::lsem.bootstrap() function can be found in Listing 5. A slight misfit is detected in fit statistics RMSEA and SRMR. The CFI and TLI fit statistics are not indicative of the incorrect invariance assumption of factor loadings.

**Figure 1:**
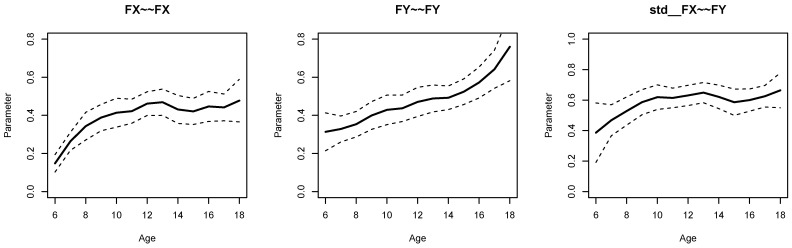
Illustrative datasets: Parameter curves for variances of the two factors (i.e., FX∼∼FX and FX∼∼FX) and the correlation of the two factors (std  FX∼∼FY) for the illustrative dataset *DATA1*.

Figure 1 displays parameter curves for the two factor variances (i.e., FX∼∼FX and FY∼∼FY) and the factor correlation (i.e., std  FX∼∼FY) for the illustrative dataset *DATA1*. From Listing 5, we see that the variance of FX had an average of 0.396 with significant parameter variation ($SD_{bc}=0.083$, p<0.001), and FY had an average of 0.473 with significant parameter variation ($SD_{bc}=0.111$, p<0.001). Moreover, the factor correlation had an average of 0.584 and also showed a significant parameter variation ($SD_{bc}=0.059$, p=0.003).

**Figure 2:**
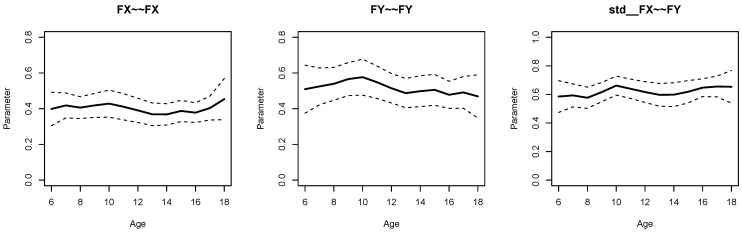
Illustrative datasets: Parameter curves for variances of the two factors (i.e., FX∼∼FX and FX∼∼FX) and the correlation of the two factors (std  FX∼∼FY) for the illustrative dataset *DATA3*.

Figure 2 displays parameter curves for the two factor variances and the factor correlation for the illustrative dataset *DATA3*, which had no simulated parameter variation in these parameters. By comparing Figure 1 and Figure 2, it is evident that there is negligible parameter variation for the dataset *DATA3* compared to the dataset *DATA1*.

**Figure 3:**
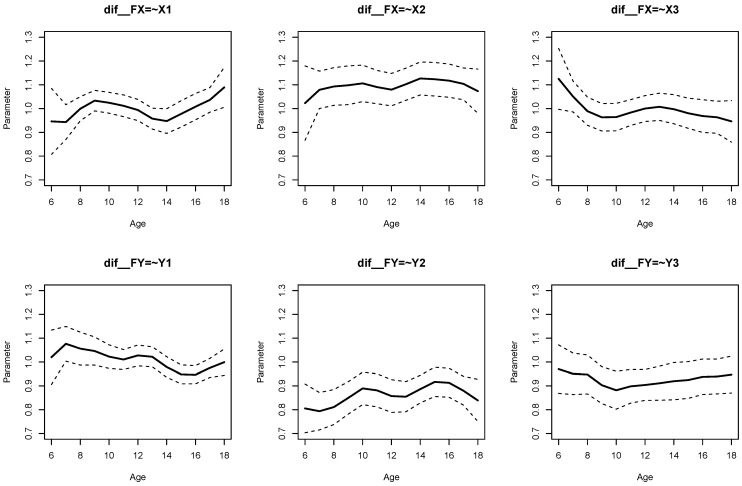
Illustrative datasets: Parameter curves for DIF effects of factor loadings for the illustrative dataset *DATA1*.

**Figure 4:**
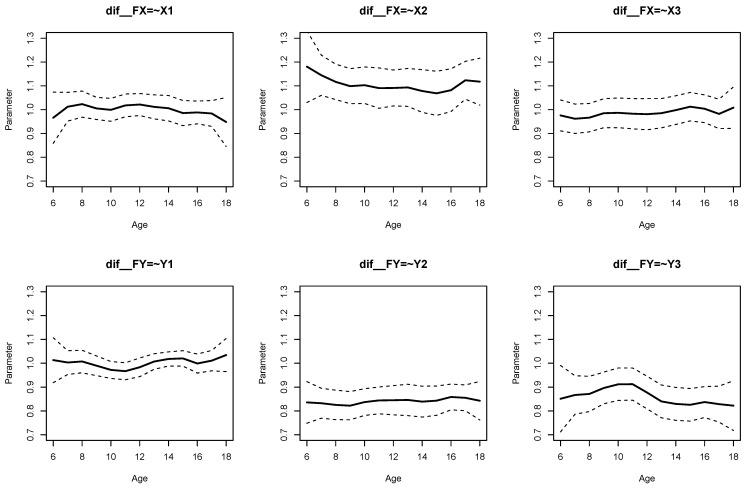
Illustrative datasets: Parameter curves for DIF effects of factor loadings for the illustrative dataset *DATA2*.

The parameter curves for DIF effects for factor loadings for datasets *DATA1* and *DATA2* are displayed in Figure 3 and Figure 4, respectively. For *DATA1*, factor loadings were simulated as noninvariant, while they were assumed invariant across age for *DATA2*. This fact is visible when comparing Figure 3 and Figure 4.

It can be seen from Listing 6 that DIF effects for factor loadings X1 ($SD_{bc}=0.024$, p=0.020), X3 ($SD_{bc}=0.038$, p=0.002), Y1 ($SD_{bc}=0.024$, p=0.022), and Y2 ($SD_{bc}=0.030$, p=0.001) had significant parameter variation for dataset *DATA1*, while they were not significant for loadings of X2 and Y3.

**Listing 6.** Illustrative datasets: Part of the output of sirt::lsem.bootstrap() for the illustrative dataset *DATA1*.

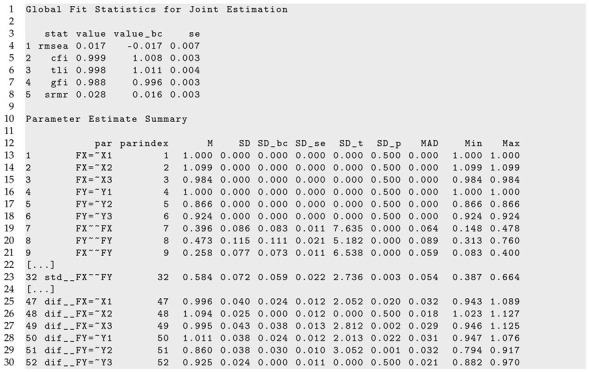



Finally, part of the R output of sirt::lsem.bootstrap() for dataset *DATA3* is displayed in Listing 7. In accordance with the data-generating model, both factor variances, the factor correlation, and the DIF effects for factor loadings did not show significant parameter variation across age.

**Listing 7.** Illustrative datasets: Part of the output of sirt::lsem.bootstrap() for the illustrative dataset *DATA3*.

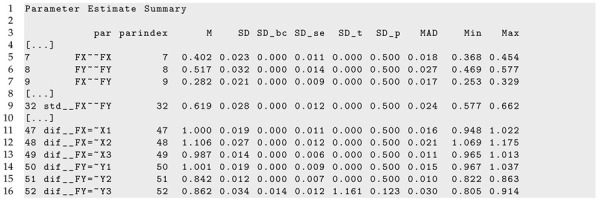



Note that a researcher will only have one dataset available for analysis. This section shows that LSEM model parameter output and figures are able to distinguish between situations of noninvariant and invariant model parameters. The standard deviation of a model parameter quantifies the variability of a model parameter across the values of the moderator.

For identification and interpretation reasons, it is useful to specify LSEM models with (some) invariant factor loadings. DIF effects reported in the LSEM output provide a post hoc assessment of the variability of parameter curves across the moderator values if parameter invariance was specified in the LSEM.

## 6. Simulation Study 1: Bias and RMSE

### 6.1. Method

In Simulation Study 1, the bias and the root mean square error (RMSE) of LSEM estimates of parameter curves were investigated. A one-factor model for three indicators, X1, X2, and X3, with a latent factor variable FX was specified. The data-generating model coincided with those from the illustrative datasets presented in Section 5. In contrast to Section 5, we only used the first three observed variables and considered a one-factor instead of a two-factor model in Simulation Study 1.

The population parameters can be found in the directory “*POPPARS*” at https://osf.io/puaz9/?view_only=63ffb2fd30f5400e89c59d03366bf793 (accessed on 3 June 2023). In this simulation, sample sizes *N* were chosen as 250, 500, 1000, 2000, and 4000. Instead of simulating data, random samples without replacement of sample size *N* were drawn from population datasets *DATA1* (noninvariant factor loadings, noninvariant factor variances and correlations), resulting in the data-generating model (DGM) DGM1, *DATA2* (invariant factor loadings, noninvariant factor variances and correlations) resulting in DGM2, and *DATA3* (invariant factor loadings, invariant factor variances and correlations), resulting in DGM3. The population datasets that included 130,000 subjects each can be found in the directory “*POPDATA*” at https://osf.io/puaz9/?view_only=63ffb2fd30f5400e89c59d03366bf793 (accessed on 3 June 2023).

Joint LSEM estimation was carried out using invariant item loadings and bandwidth factor h=1.1, 2, and 3, where the bandwidth bw was defined as $bw=hN^{-1/5}{\hat{\sigma }}_{A}$. The Gaussian kernel function was used. We also compared the two choices of computing conditional covariances with local smoothing (SM; see (17)) and the weighting approach (16) (no smoothing; NSM). Moreover, we applied LSEM with a quadratic parameter constraint (“quad”) using a bandwidth factor h=1.1. A grid of 13 focal points was chosen as 6,7,…,18.

We investigated the accuracy of the estimated parameter curves of the variance of the latent factor $F_{X}$, the invariant factor loading of the indicator $X_{2}$, and the DIF effect for factor loading of $X_{2}$. Parameter accuracy was assessed by summarizing bias and RMSE of estimated parameter curves across the different age values. The bias of a parameter $\theta (a_{t})$ at a focal point $a_{t}$ is given by:

(33) $$Bias\left(\hat{\theta }\left(a_{t}\right)\right)=\frac{1}{R}\sum_{r=1}^{R}\left({\hat{\theta }}_{r}\left(a_{t}\right)-\theta \left(a_{t}\right)\right)\;,$$

where ${\hat{\theta }}_{r}\left(a_{t}\right)$ is the parameter estimate of $\theta (a_{t})$ in the *r*th replication. The weighted absolute bias can then be defined as:

(34) $$wBias\left(\hat{\theta }\right)=\sum_{t=1}^{T}f\left(a_{t}\right)\left|Bias\left(\hat{\theta }\left(a_{t}\right)\right)\right|\;,$$

where $f(a_{t})$ denotes the proportion of values of the moderator variable that equal $a_{t}$. The weighted root mean square error (weighted RMSE) is defined as:

(35) $$wRMSE\left(\hat{\theta }\right)=\sum_{t=1}^{T}f\left(a_{t}\right)\sqrt{\frac{1}{R}\sum_{r=1}^{R}{\left({\hat{\theta }}_{r}\left(a_{t}\right)-\theta \left(a_{t}\right)\right)}^{2}}\;,$$

which is a weighted point-wise RMSE summary statistic.

In total, 2500 replications (i.e., 2500 datasets were generated and analyzed in each condition of the simulation) were conducted. We used the R (R Core Team 2023) software for the entire analysis of the simulation and the sirt (Robitzsch 2023b) package for LSEM estimation.

### 6.2. Results

In Table 1, weighted absolute bias and weighted RMSE for the factor variance, the invariant factor loading of $X_{2}$, and the DIF effect of factor loading of $X_{2}$ are presented.

It turned out that all three model parameters resulted in unbiased estimation for moderate or large sample sizes. For DGM1 or DGM2, the quadratic parameter constraint introduced some misspecification, which led to slight biases. Moreover, using the local quadratic smoothing approach SM for estimating conditional covariances instead of the weighted approach NM (e.g., no smoothing) resulted in a small error bias. Finally, biases increased with increasing the bandwidth factor *h*.

Notably, using local smoothing SM for conditional covariances added variability in terms of RMSE compared to NM. Regarding RMSE, one could conclude that h=2 seems preferable to h=1.1 or h=3 (see also Hildebrandt et al. 2016).

Overall, the findings of Simulation Study 1 demonstrated that joint LSEM estimation resulted in approximately unbiased parameter estimates. The decrease in RMSE values for increasing sample sizes also indicated that parameter estimates are consistent. Notably, the recommendation of using the bandwidth factor h=2 in pointwise LSEM (Hildebrandt et al. 2016) also transfers to the joint LSEM estimation method.

## 7. Simulation Study 2: Estimation of Variability of Model Parameters and Statistical Significance Tests

### 7.1. Method

In Simulation Study 2, the bias of standard deviation statistics for parameter variation and the properties of significance tests for parameter variation are investigated. The same three data-generating models DGM1, DGM2, and DGM3 as in Simulation Study 1 (see Section 6.1) were utilized.

The chosen sample sizes in this simulation were N=500, 1000, 2000, and 4000. As in Simulation Study 1, samples of sample size *N* were drawn without replacement from population datasets *DATA1*, *DATA2*, and *DATA3* for DGM1, DGM2, and DGM3, respectively. The population datasets that included 130,000 subjects can be found in the directory “*POPDATA*” at https://osf.io/puaz9/?view_only=63ffb2fd30f5400e89c59d03366bf793 (accessed on 3 June 2023).

As in Simulation Study 1, a one-factor model with indicators X1, X2, and X3 was specified. Throughout all simulation conditions, a bandwidth factor of h=2 was chosen. The bias of the two standard deviation estimators ${\hat{\mathrm{SD}}}_{\theta (a)}$ and ${\hat{\mathrm{SD}}}_{\theta (a),\mathrm{bc}}$ defined in (28) and (29) was assessed. Significance testing for parameter variation was based on the standard deviation (see (30)), which uses a normal distribution approximation and the Wald test (see (31)), which uses a chi-square distribution as a null distribution. Statistical significance tests were performed with significance levels of 0.05 and 0.01. The bias of the standard deviation variability statistics and significance tests of parameter variation was computed for the variance of the latent factor, the three DIF effects of the factor loadings, and the three residual variances.

In total, 2500 replications were conducted in all simulation conditions. The R software (R Core Team 2023) was used for analyzing this simulation study, and the R package sirt (Robitzsch 2023b) was employed for LSEM estimation and significance testing.

### 7.2. Results

In Table 2, the bias of raw and bias-corrected (“bc”) estimates of the standard deviation variability measure ${\mathrm{SD}}_{\theta (a)}$ are presented. In DGM1, all parameters have nonvanishing ${\mathrm{SD}}_{\theta (a)}$ values for the population dataset *DATA1*. In this case, the raw SD estimate showed some slight positive bias for sample sizes N=500 and 1000. The bias-corrected estimates were generally negatively biased, although the biases were not very large. In DGM2, only the variance of the latent factor *F* had a true parameter variation larger than 0. In this situation, raw estimates were approximately unbiased, while the bias-corrected estimates were negatively biased. If there was no true parameter variation, such as for DIF effects or residual variances in DGM2 or all parameters in DGM3, the bias-corrected estimates were less biased than the raw standard deviation estimate.

Overall, one could say that for smaller values of true variability, the positive bias in the raw SD estimate was larger than the underestimation of the bias-corrected SD estimate. An improved SD statistic might be obtained by computing some weighted average of the raw and the bias-corrected estimate.

Table 3 presents type I error and power rates for the different LSEM model parameters. Significance testing based on the SD statistics had inflated type I error rates. If the nominal level was chosen as 1%, the empirical error rate was about 5%. Moreover, the Wald statistic had type I error rates lower than the nominal level in many simulation conditions. Nevertheless, significance testing based on the standard deviation has substantially more statistical power. If a target nominal significance level for the SD test statistic were 5%, it is advised to use a significance level of 0.01.

## 8. Empirical Example: A Reanalysis of SON-R

### 8.1. Data

According to the age differentiation hypothesis, cognitive abilities become more differentiated with increasing age during childhood. Hülür et al. (2011) used data from the German standardization of the SON-R 212−7 intelligence test to examine age-related differentiation of cognitive abilities from age 212 to age 7. The SON-R 212−7 intelligence test is a nonverbal intelligence test for children and consists of six indicators (i.e., six subtests). The SON-R 212−7 test contains two subscales measured by three indicators each. The performance subscale (with factor Fp) contains indicators mosaics (p1), puzzles (p2), and patterns (p3). The reasoning subscale (with factor Fr) contains the indicators categories (r1), analogies (r2), and situations (r3).

Unfortunately, the SON-R dataset is not publicly available, and the authors of this paper cannot publicly share the dataset on the internet. To replicate the LSEM analysis of this example, we generated a synthetic dataset of the SON-R 212−7 data based on the original dataset used in Hülür et al. (2011). The same sample size of N=1027 children was simulated. In the synthetic data generation, we relied on a recently proposed method by Jiang et al. (2021) (see also Grund et al. 2022, Nowok et al. 2016 or Reiter 2023) that combines the distinct approaches of simulating a dataset based on a known distribution and the approach of adding to noise to original data to prevent data disclosure or person identification. The noisy versions of the original dataset were simulated with a reliability of 0.95 (Grund et al. 2022), and quadratic relations among variables were allowed. The data synthesis model was separately carried out in 18 groups of children (i.e., in 9 age groups for male and female children, respectively). The values of the age and gender variables were held fixed in the analysis meaning that these demographic variables had the same distribution in the synthetic data as in the original data. In total, 50.8% of the children in the sample was male. The synthetic data and syntax for synthetic data generation can be found in the directory “*SON-R*” at https://osf.io/puaz9/?view_only=63ffb2fd30f5400e89c59d03366bf793 (accessed on 3 June 2023).

The indicator variables were linearly transformed such that the mean equaled zero and the standard deviation equaled one for children aged between 4.0 and 6.0 years. This is an arbitrary choice and only affects the scaling of the variables. The assessment of model parameter heterogeneity in the LSEM is independent of this choice. Alternatively, one might also standardize the indicator variables for children in the total sample with ages between 2.5 and 7.5 years.

A two-dimensional CFA model involving the performance and the reasoning factor was specified in an LSEM analysis. The mean structure remained unmodeled because the primary goal of this analysis was to investigate the age differentiation hypothesis. For model identification, the factor loadings were assumed as invariant across age, and the first loading of both scales (i.e., loadings of p1 and r1) were fixed at one. In accordance with Hildebrandt et al. (2016) and the findings of Simulation Study 1, the bandwidth factor of h=2 was chosen, resulting in a bandwidth $bw=2N^{-1/5}{\hat{\sigma }}_{A}$, where ${\hat{\sigma }}_{A}=1.23$ is the estimated standard deviation of the age variable. Because the LSEM model involved invariance constraints among parameters, a joint estimation approach was employed. For statistical inference and the test of parameter variation, R=200 bootstrap samples were drawn. Replication syntax can also be found in the directory “*SON-R*” at https://osf.io/puaz9/?view_only=63ffb2fd30f5400e89c59d03366bf793 (accessed on 3 June 2023).

### 8.2. Results

**Figure 5:**
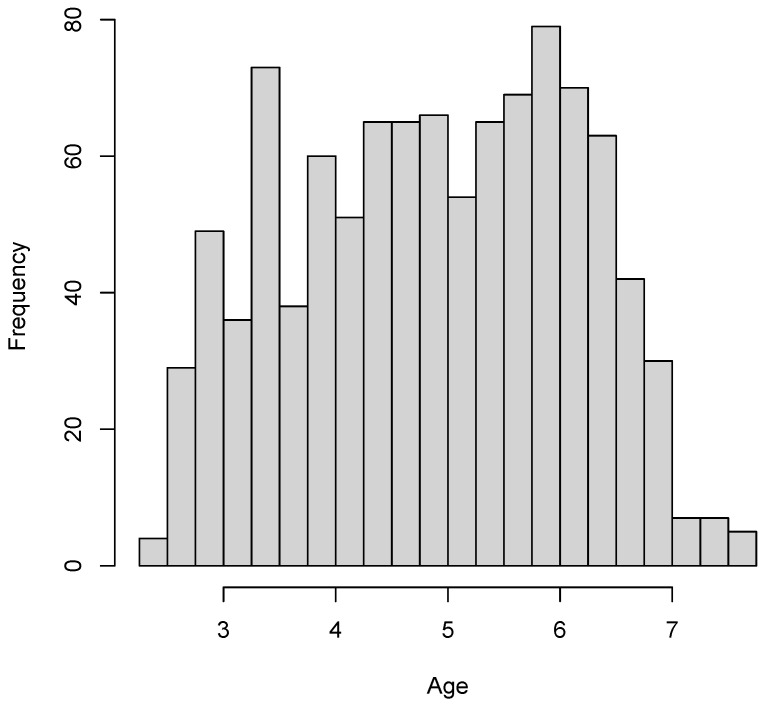
SON-R example: Histogram for moderator age.

Figure 5 displays the histogram of the age variable. The age of children ranged between 2.44 and 7.72 years, with a mean of 4.89 and a standard deviation of 1.23. The histogram indicated that the intended age range between 2.5 and 7 years of the SON-R 212−7 test was approximately uniformly distributed.

The estimated LSEM model had an acceptable model fit regarding typical model fit effect sizes. The fit statistics without bias correction were RMSEA = 0.061, CFI = 0.952, TLI = 0.960, GFI = 0.963, and SRMR = 0.055.

In Listing 8, parts of the LSEM output of lsem.bootstrap() are displayed. According to the specified model, the parameter variation (i.e., SD and SD_bc) for factor loadings (i.e., Fp=∼p1, …, F3=∼r3) was zero because the parameters were assumed invariant across age.

**Listing 8.** SON-R example: Part of the output of lsem.bootstrap() function.

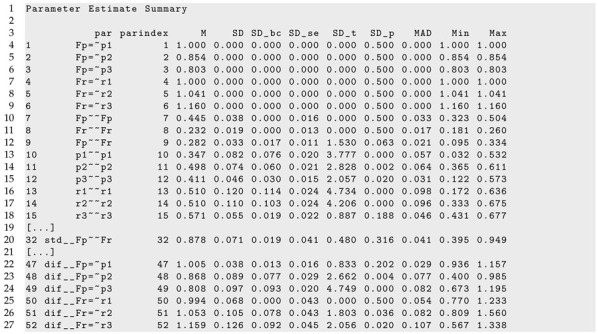



**Figure 6:**
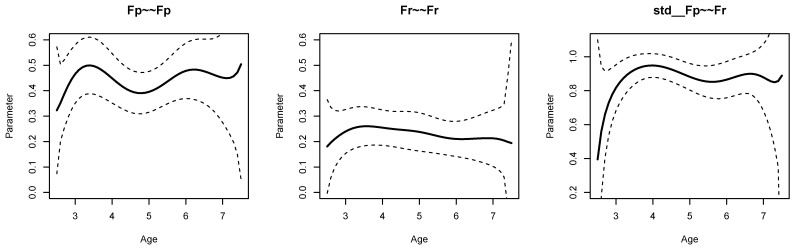
SON-R example: Parameter curves for variances of performance (Fp∼∼Fp) and reasoning (Fr∼∼Fr) and the correlation of performance and reasoning (std  Fp∼∼Fr).

The age differentiation hypothesis refers to the variances of the performance scale (i.e., Fp∼∼Fp), the variance of the reasoning scale (i.e., Fr∼∼Fr), and the correlation of both factors (i.e., std  Fp∼∼Fr). Figure 6 displays the parameter curves with confidence intervals for the two variances and the correlation. From the R output presented in Listing 8, it can be seen that the variances parameter curves did not show significant parameter variation, and the bias-corrected standard deviation estimate SD_bc was 0.000. The correlation between the performance and the reasoning scale was 0.878 on average, with a small bias-corrected standard deviation estimate of 0.019 that turned out to be nonsignificant (*p* = 0.316). Hence, there was no evidence for the age differentiation hypothesis in the SON-R dataset.

**Figure 7:**
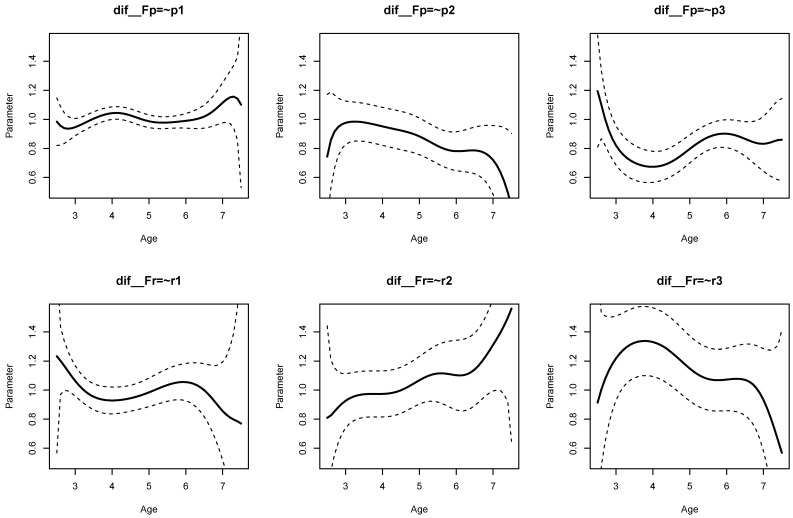
SON-R example: Parameter curves for DIF effects of factor loadings for performance (latent variable Fp) and reasoning (latent variable Fr).

Figure 7 displays the parameter curves of the DIF effects of the factor loadings. The corresponding parameters for DIF effects can be found in lines 47 to 52 in Listing 8 (i.e., parameters dif  Fp=∼p1, …, dif  Fr=∼r3). There was substantial parameter variation in terms of the bias-corrected standard deviation SD_ bc for the loadings of p2 ($SD_{bc}=0.077$, p=0.004), p3 ($SD_{bc}=0.077$, p<0.001), r2 ($SD_{bc}=0.078$, p=0.036), and r3 ($SD_{bc}=0.092$, p=0.020).

**Figure 8:**
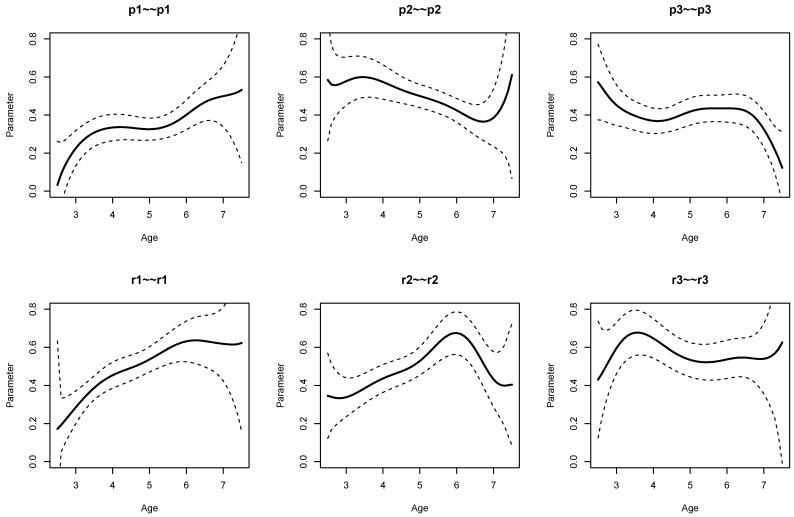
SON-R example: Parameter curves for residual variances.

Finally, residual variances are displayed in Figure 8. From the results from Listing 8, it is evident that residual variances of p1 ($SD_{bc}=0.076$, p<0.001), p2 ($SD_{bc}=0.060$, p=0.002), r1 ($SD_{bc}=0.114$, p<0.001), and r2 ($SD_{bc}=0.103$, p<0.001) were statistically significant at the 0.01 significance level.

Note that Hülür et al. (2011) used a pointwise LSEM approach instead a joint LSEM estimation approach. The identification of parameters in the covariance structure of factors was achieved in Hülür et al. (2011) by the constraint that the pointwise average of factor loadings equaled 1. Due to the different estimation approaches, it is expected that there are slight differences between our joint LSEM estimation approach and the original analysis in Hülür et al. (2011). The parameter curve of the correlation between the performance and the reasoning factors was similar in both analyses, with the exception that the factor correlation for small age values was much lower in the joint estimation approach, as displayed in Figure 6.

An anonymous reviewer wondered whether the factor correlation could be meaningfully interpreted if factor loadings did not show invariance across the moderator values. We argued elsewhere that measurement invariance would be a helpful but not a necessary condition for a meaningful interpretation of a factor correlation or a factor variance (see Robitzsch and Lüdtke 2023). In fact, a violation of measurement invariance only implies that results would change if a subset of indicators was used in the factor model. Because the SON-R instrument is held fixed in test administration and statistical analysis, this property of item selection invariance is not required. Of course, any identification constraint on factor loadings must be imposed to identify a factor correlation. The choice of identification constraint is somehow arbitrary. It could be invariance of all factor loadings, invariance of loadings of a subset of indicators, or a pointwise constraint of the average loadings (i.e., the average loading should be 1 for all indicators of a factor).

## 9. Discussion

In this article, we discussed the implementation of LSEM in the R package sirt. Joint LSEM estimation and two different significance tests for a test of parameter variation were introduced and evaluated through two simulation studies.

Simulation Study 1 demonstrated that the joint LSEM estimation method can be successfully applied to structural equation models whose parameters vary across different values of the moderator variable. It turned out that the bandwidth factor h=2 can generally be recommended as a default choice. Notably, LSEM model parameters can be quite variable for small (N=250) or moderate sample sizes (N=500). In Simulation Study 2, two significance testing approaches for constant parameter curves were investigated: a test statistic based on the standard deviation of a parameter curve and a Wald-type test statistic. Both testing approaches rely on bootstrap samples for statistical inference. The standard-deviation-based test statistics had a higher power than the Wald test-type test statistic, but also came with an inflated type-I error rate. It is recommended to use the significance test based on the standard deviation with a significance level of 1% if a nominal significance level of 5% is required.

The application of LSEM in applied research can be regarded more as an exploratory than a confirmatory statistical method (Jacobucci 2022). Functional forms of parameter curves obtained with LSEM can be validated in other samples or future studies with more confirmatory approaches, such as moderated nonlinear factor analysis. We would like to emphasize that sufficiently large sample sizes are required in LSEM in order to allow a reliable interpretation of the obtained nonlinear parameter curves. Moreover, the true variability in parameter curves must be sufficiently large to have enough power to statistically detect the significant parameter variability. A statistical significance test on parameter curve regression coefficients in a moderated nonlinear factor analysis might have more power than a test based on the nonparametric LSEM method. Finally, moderated nonlinear factor analysis, if estimated by maximum likelihood, allows likelihood ratio tests for testing among nested models or using information criteria for model comparisons.

In this article, the moderator variable was exclusively age and a bounded variable. There might be applications in which the moderator differs from age, such as unbounded self-concept factor variables or ability values obtained from item response models (Basarkod et al. 2023). Because the metric of such variables is often arbitrary, it is advised to transform such moderators into a bounded metric. For example, the percentage ranks of an unbounded moderator variable could be utilized to obtain a bounded moderator variable.

If the moderator variable is an error-prone variable such as a factor variable or a scale score, an expected a posteriori (EAP) factor score estimate can be used as a moderator to obtain unbiased estimates of LSEM model parameters (Bartholomew et al. 2011).

As explained in Section 4, datasets with missing values should either be handled with pairwise deletion methods for computing sufficient statistics (i.e., the conditional covariance matrices) in LSEM or should be multiply imputed. The imputation model should be flexibly specified to represent the complex associations modeled with LSEM. For example, the moderator variable could be discretized into 5 or 10 distinct groups, and the resulting datasets should be separately imputed in the separate subdatasets. Statistical inference should be carried out that involves the multiply imputed datasets (Little and Rubin 2002).

Finally, we only discussed LSEM in the case of one moderator variable. With more than one moderator variable (Hartung et al. 2018), moderated nonlinear factor analysis might be easier to estimate because multivariate kernel functions for LSEM are difficult to estimate with sparse data.

## Figures and Tables

**Table 1:** Simulation Study 1: Weighted absolute bias and weighted root mean square error (RMSE) for the parameter curve θ(a) for different model parameters as a function of sample size *N* and three data-generating models DGM1, DGM2 and DGM3.

		Weighted Absolute Bias							Weighted RMSE						
		h=1.1		h=2		h=3			h=1.1		h=2		h=3		
DGM	N	SM	NSM	SM	NSM	SM	NSM	Quad	SM	NSM	SM	NSM	SM	NSM	Quad
		*Variance of latent factor F*													
1	250	0.022	0.022	0.021	0.028	0.025	0.039	0.023	0.088	0.083	0.078	0.076	0.077	0.079	0.076
	500	0.013	0.014	0.015	0.022	0.020	0.033	0.016	0.064	0.061	0.057	0.056	0.056	0.060	0.054
	1000	0.007	0.009	0.010	0.018	0.016	0.029	0.011	0.048	0.046	0.042	0.043	0.042	0.048	0.039
	2000	0.006	0.008	0.008	0.015	0.013	0.025	0.010	0.035	0.034	0.031	0.033	0.032	0.038	0.029
	4000	0.003	0.005	0.005	0.012	0.010	0.021	0.008	0.026	0.026	0.023	0.025	0.023	0.030	0.021
2	250	0.023	0.019	0.021	0.022	0.022	0.030	0.018	0.086	0.080	0.076	0.070	0.073	0.070	0.073
	500	0.013	0.011	0.014	0.017	0.017	0.026	0.011	0.063	0.059	0.055	0.053	0.054	0.054	0.052
	1000	0.008	0.008	0.010	0.015	0.014	0.023	0.008	0.046	0.044	0.040	0.040	0.040	0.042	0.038
	2000	0.005	0.006	0.007	0.012	0.012	0.020	0.006	0.034	0.033	0.030	0.030	0.029	0.032	0.027
	4000	0.003	0.004	0.005	0.009	0.009	0.017	0.005	0.025	0.025	0.022	0.023	0.022	0.026	0.020
3	250	0.018	0.017	0.012	0.010	0.009	0.007	0.017	0.087	0.080	0.076	0.067	0.071	0.061	0.073
	500	0.010	0.009	0.006	0.005	0.004	0.004	0.009	0.063	0.059	0.055	0.049	0.051	0.044	0.052
	1000	0.006	0.006	0.004	0.004	0.003	0.003	0.006	0.047	0.044	0.040	0.036	0.037	0.033	0.038
	2000	0.002	0.002	0.001	0.001	0.001	0.001	0.002	0.034	0.032	0.029	0.026	0.026	0.023	0.026
	4000	0.002	0.002	0.001	0.001	0.001	0.001	0.002	0.025	0.024	0.021	0.020	0.019	0.017	0.019
		*Invariant factor loading of $X_{2}$*													
1	250	0.003	0.005	0.006	0.004	0.008	0.004	0.005	0.089	0.090	0.089	0.089	0.090	0.088	0.089
	500	0.001	0.001	0.000	0.000	0.002	0.000	0.001	0.063	0.063	0.062	0.063	0.063	0.062	0.063
	1000	0.001	0.000	0.001	0.000	0.001	0.000	0.000	0.043	0.043	0.043	0.043	0.044	0.043	0.043
	2000	0.001	0.002	0.000	0.002	0.002	0.001	0.001	0.031	0.031	0.030	0.031	0.031	0.031	0.031
	4000	0.000	0.001	0.001	0.001	0.000	0.000	0.000	0.022	0.022	0.022	0.022	0.022	0.022	0.022
2	250	0.005	0.005	0.004	0.004	0.004	0.004	0.005	0.091	0.091	0.091	0.090	0.091	0.090	0.091
	500	0.002	0.002	0.002	0.002	0.002	0.002	0.002	0.062	0.062	0.062	0.062	0.062	0.061	0.062
	1000	0.001	0.001	0.001	0.001	0.001	0.001	0.001	0.045	0.045	0.045	0.045	0.045	0.045	0.045
	2000	0.001	0.001	0.001	0.001	0.001	0.001	0.001	0.031	0.031	0.031	0.031	0.031	0.031	0.031
	4000	0.000	0.000	0.000	0.000	0.000	0.000	0.000	0.022	0.022	0.022	0.022	0.022	0.022	0.022
3	250	0.003	0.003	0.002	0.003	0.002	0.003	0.003	0.090	0.090	0.090	0.090	0.090	0.090	0.090
	500	0.003	0.003	0.003	0.003	0.003	0.002	0.003	0.064	0.064	0.063	0.064	0.064	0.064	0.064
	1000	0.000	0.001	0.000	0.001	0.000	0.001	0.001	0.043	0.043	0.043	0.043	0.043	0.044	0.043
	2000	0.000	0.000	0.000	0.000	0.000	0.000	0.000	0.032	0.032	0.032	0.032	0.032	0.032	0.032
	4000	0.000	0.000	0.000	0.000	0.000	0.000	0.000	0.022	0.022	0.022	0.022	0.022	0.022	0.022
		*DIF for factor loading of $X_{2}$*													
1	250	0.007	0.007	0.010	0.012	0.013	0.015	0.013	0.123	0.109	0.111	0.098	0.105	0.094	0.123
	500	0.004	0.006	0.008	0.011	0.012	0.014	0.013	0.085	0.079	0.078	0.071	0.075	0.068	0.092
	1000	0.003	0.004	0.006	0.009	0.010	0.013	0.012	0.061	0.057	0.055	0.051	0.053	0.049	0.069
	2000	0.002	0.004	0.005	0.008	0.008	0.012	0.012	0.044	0.041	0.040	0.037	0.038	0.036	0.053
	4000	0.002	0.003	0.004	0.007	0.007	0.010	0.012	0.032	0.031	0.029	0.027	0.028	0.027	0.041
2	250	0.005	0.005	0.005	0.004	0.004	0.004	0.009	0.121	0.110	0.109	0.098	0.105	0.094	0.122
	500	0.002	0.002	0.002	0.002	0.002	0.002	0.008	0.083	0.077	0.075	0.069	0.072	0.065	0.089
	1000	0.001	0.001	0.001	0.001	0.001	0.001	0.008	0.061	0.058	0.055	0.051	0.053	0.048	0.068
	2000	0.001	0.001	0.001	0.001	0.001	0.001	0.007	0.043	0.041	0.039	0.036	0.037	0.034	0.051
	4000	0.000	0.000	0.000	0.000	0.000	0.000	0.006	0.032	0.030	0.028	0.026	0.027	0.025	0.039
3	250	0.003	0.003	0.003	0.003	0.002	0.003	0.002	0.118	0.109	0.106	0.098	0.102	0.094	0.120
	500	0.003	0.003	0.003	0.003	0.003	0.003	0.002	0.083	0.079	0.076	0.071	0.073	0.067	0.090
	1000	0.001	0.001	0.001	0.001	0.001	0.001	0.001	0.059	0.056	0.053	0.050	0.050	0.047	0.066
	2000	0.000	0.000	0.000	0.000	0.000	0.000	0.001	0.044	0.042	0.039	0.037	0.037	0.035	0.051
	4000	0.000	0.000	0.000	0.000	0.000	0.000	0.001	0.031	0.030	0.028	0.026	0.026	0.025	0.038

**Table 2:** Simulation Study 2: Bias of raw and bias-corrected estimators of the standard deviation ${\mathrm{SD}}_{\theta (a)}$ for the parameter curve θ(a) for different model parameters as a function of sample size *N* and three data-generating models DGM1, DGM2 and DGM3.

	DGM 1			DGM 2			DGM 3		
	${\mathrm{SD}}_{\theta (a)}$			${\mathrm{SD}}_{\theta (a)}$			${\mathrm{SD}}_{\theta (a)}$		
N	true	raw	bc	true	raw	bc	true	raw	bc
	*Variance of latent factor F*								
500	−0.081	−0.002	−0.012	−0.054	−0.002	−0.013	0	−0.035	−0.013
1000	−0.081	−0.003	−0.009	−0.054	−0.001	−0.009	0	−0.027	−0.010
2000	−0.081	−0.003	−0.006	−0.054	−0.002	−0.006	0	−0.020	−0.007
4000	−0.081	−0.003	−0.005	−0.054	−0.002	−0.004	0	−0.015	−0.005
	*DIF for factor loading of $X_{1}$*								
500	−0.047	−0.007	−0.021	0	−0.041	−0.013	0	−0.039	−0.013
1000	−0.047	−0.001	−0.017	0	−0.031	−0.010	0	−0.029	−0.010
2000	−0.047	−0.002	−0.012	0	−0.023	−0.007	0	−0.022	−0.007
4000	−0.047	−0.002	−0.008	0	−0.017	−0.005	0	−0.017	−0.005
	*DIF for factor loading of $X_{2}$*								
500	−0.021	−0.021	−0.007	0	−0.038	−0.012	0	−0.036	−0.012
1000	−0.021	−0.013	−0.007	0	−0.029	−0.009	0	−0.027	−0.009
2000	−0.021	−0.006	−0.008	0	−0.022	−0.007	0	−0.021	−0.007
4000	−0.021	−0.003	−0.007	0	−0.017	−0.005	0	−0.016	−0.005
	*DIF for factor loading of $X_{3}$*								
500	−0.022	−0.009	−0.008	0	−0.022	−0.006	0	−0.021	−0.006
1000	−0.022	−0.005	−0.006	0	−0.017	−0.005	0	−0.016	−0.005
2000	−0.022	−0.002	−0.004	0	−0.013	−0.004	0	−0.012	−0.004
4000	−0.022	−0.001	−0.002	0	−0.009	−0.003	0	−0.009	−0.003
	*Residual variance of $X_{1}$*								
500	−0.012	−0.014	−0.001	0	−0.024	−0.009	0	−0.024	−0.009
1000	−0.012	−0.009	−0.003	0	−0.018	−0.006	0	−0.018	−0.006
2000	−0.012	−0.005	−0.003	0	−0.014	−0.005	0	−0.014	−0.005
4000	−0.012	−0.003	−0.003	0	−0.011	−0.003	0	−0.011	−0.003
	*Residual variance of $X_{2}$*								
500	−0.007	−0.020	−0.003	0	−0.027	−0.010	0	−0.027	−0.010
1000	−0.007	−0.014	−0.001	0	−0.021	−0.007	0	−0.020	−0.007
2000	−0.007	−0.010	−0.001	0	−0.016	−0.005	0	−0.016	−0.005
4000	−0.007	−0.006	−0.002	0	−0.012	−0.004	0	−0.012	−0.004
	*Residual variance of $X_{3}$*								
500	−0.011	−0.009	−0.002	0	−0.018	−0.007	0	−0.018	−0.007
1000	−0.011	−0.006	−0.002	0	−0.014	−0.005	0	−0.014	−0.005
2000	−0.011	−0.003	−0.003	0	−0.010	−0.003	0	−0.011	−0.004
4000	−0.011	−0.002	−0.002	0	−0.008	−0.002	0	−0.008	−0.003

true = true value of ${\mathrm{SD}}_{\theta (a)}$ in infinite sample size (i.e., at the population level); raw = raw estimate ${\hat{\mathrm{SD}}}_{\theta (a)}$ of ${\mathrm{SD}}_{\theta (a)}$ (see Equation (28)); bc = bias-corrected estimate ${\hat{\mathrm{SD}}}_{\theta (a),\mathrm{bc}}$ of ${\mathrm{SD}}_{\theta (a)}$ (see Equation (29)).

**Table 3:** Simulation Study 2: Type I and power rates for the significance test for variability in a parameter curve θ(a) for the two test statistics based on ${\mathrm{SD}}_{\theta (a)}$ (SD) and the Wald test (WA) as a function of sample size *N* and three data-generating models DGM1, DGM2 and DGM3.

	DGM1				DGM2				DGM3			
N	SD5	WA5	SD1	WA1	SD5	WA5	SD1	WA1	SD5	WA5	SD1	WA1
	*Variance of latent factor F*											
500	92.4	46.9	79.8	29.6	66.0	17.8	44.2	8.6	16.3	1.6	4.9	0.5
1000	99.7	88.1	98.5	75.4	90.0	45.7	76.1	26.5	17.4	2.8	5.9	0.8
2000	100.0	99.8	100.0	99.1	99.1	83.0	97.0	65.8	17.7	3.6	5.8	0.9
4000	100.0	100.0	100.0	100.0	100.0	99.5	100.0	98.1	18.3	6.4	7.7	2.0
	*DIF for factor loading of $X_{1}$*											
500	23.7	1.3	8.2	0.4	12.9	0.3	3.4	0.1	13.2	0.4	3.4	0.0
1000	46.1	6.0	22.8	1.2	14.8	0.8	5.2	0.2	14.7	0.5	5.1	0.1
2000	75.2	25.2	52.1	10.7	14.4	1.6	4.9	0.3	15.9	1.7	5.5	0.2
4000	96.2	70.6	89.6	48.7	17.5	4.0	6.1	1.1	17.9	4.1	7.1	1.1
	*DIF for factor loading of $X_{2}$*											
500	12.3	0.4	3.3	0.1	12.4	0.3	3.5	0.0	12.3	0.3	3.3	0.0
1000	21.7	2.2	8.6	0.5	13.1	0.7	4.1	0.1	13.9	0.8	4.7	0.1
2000	31.2	5.3	14.9	1.5	16.7	1.8	5.8	0.4	16.3	1.4	5.3	0.4
4000	52.4	19.1	32.1	8.2	16.2	3.6	6.9	0.7	18.1	4.2	7.1	1.3
	*DIF for factor loading of $X_{3}$*											
500	18.4	0.4	5.4	0.1	7.5	0.2	1.6	0.0	8.3	0.1	1.8	0.0
1000	38.6	2.0	16.5	0.4	10.5	0.4	2.5	0.0	12.1	0.3	2.9	0.0
2000	66.1	13.7	42.5	4.6	14.2	0.8	4.6	0.0	13.8	1.0	4.6	0.2
4000	92.3	52.0	81.0	28.8	16.1	2.3	5.8	0.5	17.7	2.8	6.2	0.4
	*Residual variance of $X_{1}$*											
500	20.7	2.3	7.5	0.9	18.2	2.2	6.8	0.5	16.6	2.0	6.2	0.7
1000	25.3	4.0	10.4	1.6	16.6	2.8	5.6	0.9	17.8	3.1	6.5	0.8
2000	34.7	8.4	17.4	2.6	18.3	4.4	6.4	1.2	18.0	4.3	7.1	1.0
4000	49.1	17.7	29.1	7.1	17.3	5.7	7.0	1.5	19.0	6.0	7.2	1.6
	*Residual variance of $X_{2}$*											
500	16.9	1.9	5.9	0.4	17.7	1.9	6.4	0.4	17.1	1.8	6.3	0.6
1000	18.7	2.5	7.0	0.7	17.6	2.8	6.2	0.7	17.8	2.8	5.7	0.9
2000	22.1	4.8	8.4	1.3	18.5	4.4	7.1	1.3	16.7	3.6	5.9	1.0
4000	29.6	8.8	13.3	3.0	18.0	5.9	7.4	1.6	19.0	6.5	7.1	1.6
	*Residual variance of $X_{3}$*											
500	25.4	2.9	11.2	0.8	16.7	1.4	5.6	0.5	17.7	2.0	6.1	0.5
1000	34.1	5.5	15.9	1.5	17.9	2.7	6.2	0.7	18.1	2.7	6.4	0.8
2000	47.3	12.0	26.6	4.8	17.2	3.3	6.4	0.8	18.3	4.0	6.7	1.1
4000	68.6	26.3	47.4	12.7	17.9	5.3	7.0	1.4	19.2	6.5	7.7	1.7

*Note*. SD5 = test statistic based on bias-corrected ${\mathrm{SD}}_{\theta (a)}$ estimate at 5% confidence level; WA5 = Wald test statistic at 5% confidence level; SD1 = test statistic based on bias-corrected ${\mathrm{SD}}_{\theta (a)}$ estimate at 1% confidence level; WA1 = Wald test statistic at 1% confidence level; Cells with yellow-gray colored background correspond to type I error rates, while cells with white background color correspond to power rates.

## Data Availability

Datasets and R code is available as supplementary material at https://osf.io/puaz9/?view_only=63ffb2fd30f5400e89c59d03366bf793 (accessed on 3 June 2023).

## Abbreviations

The following abbreviations are used in this manuscript:

CFA	confirmatory factor analysis
DGM	data-generating model
DIF	differential item functioning
LSEM	local structural equation model
ML	maximum likelihood
MNFA	moderated nonlinear factor analysis
SEM	structural equation model
